# Gunsight sutures significantly reduce surgical-site infection after ileostomy reversal compared with linear sutures

**DOI:** 10.1093/gastro/goaa075

**Published:** 2020-12-10

**Authors:** Chuang-Kun Li, Wei-Wen Liang, Huai-Ming Wang, Wen-Tai Guo, Xiu-Sen Qin, Jie Zhao, Wen-Bin Zhou, Yang Li, Hui Wang, Rong-Kang Huang

**Affiliations:** 1 Department of Colorectal Surgery, the Sixth Affiliated Hospital, Sun Yat-sen University, Guangzhou, Guangdong, P. R. China; 2 Guangdong Provincial Key Laboratory of Colorectal and Pelvic Floor Diseases, The Sixth Affiliated Hospital of Sun Yat-sen University, Guangzhou, Guangdong, P. R. China

**Keywords:** ileostomy reversal, linear suture, gunsight suture, surgical-site infection

## Abstract

**Background:**

Surgical-site infection (SSI) was one of the most common post-operative morbidities of ileostomy reversal. Although several skin-closure procedures had been developed to reduce the rate of SSI, the optimal procedure remains unclear. In this study, we compared the effect of two surgical techniques for wound closure following ileostomy reversal: gunsight suture (GS) and linear suture (LS).

**Methods:**

A total of 233 patients who underwent loop ileostomy at the Sixth Affiliated Hospital of Sun Yat-sen University between January 2015 and December 2017 were enrolled into our study. These patients were divided into two groups: the LS group and the GS group. We compared the clinical characteristics between the two groups and analyzed the data using IBM SPSS to identify risk factors for SSI.

**Results:**

Both groups successfully underwent surgery. The rate of SSI was significantly lower in the GS group (*n* = 2, 0.02%) than in the LS group (*n* = 16, 12.00%, *P* = 0.007). The length of hospital stay after the operation in the GS group was significantly shorter than that in the LS group (8.1 ± 3.2 vs 10.8 ± 5.4 days, *P* < 0.001). Multivariate analysis showed that GS was an independent protective risk factor for SSI (odds ratio = 0.212, *P* = 0.048).

**Conclusions:**

Compared with the LS technique, the GS technique can significantly decrease the rate of SSI and shorten the length of hospital stay after surgery. The GS technique may be recommended for wound closure following ileostomy reversal.

## Introduction

Diverting ileostomy reversal is susceptible to many complications, among them surgical-site infection (SSI) was the most common one [1]. SSI could lead to prolonged hospital stay, higher costs for wound care, and an unaesthetic appearance as the wound heals. To decrease the rate of SSI, different skin-closure techniques had been reported in the literature. Previously, the linear suture (LS) was the most common method for wound closure and had a reported SSI rate ranging from 3% to 41% [2]. Many other wound-closure methods had been postulated to reduce the risk of SSI [3]. In 1997, Banerjee [4] introduced a purse-string skin-closure technique and showed a significantly reduced risk of SSI compared with those reported in the conventional LS group [5, 6]. However, the small incisions did not provide good exposure for dissection for the bowel and the purse-string suture [7, 8] had a prolonged wound-healing time because of the 5- to 10-mm gap that remains after reversal, leaving a scar with a puckered appearance. Then, Lim *et al.* [9] introduced a novel skin-closure technique named the gunsight suture (GS). In addition, the patients who underwent GS had a low rate of SSI and there was satisfactory drainage at the surgical skin site after GS [10, 11]. The results of a multicenter prospective randomized trial have shown that the GS and purse-string suture had similar rates of SSI, could be effectively applied for skin closure after stoma reversal, and GS had a shorter incision healing time compared with the purse-string suture [12]. However, the optimal skin-closure technique has not yet been established due to the limited cases reported to date. Our retrospective study was conducted to compare the effect of two skin-closure techniques (LS and GS) on SSI.

## Patients and methods

### Patients

We included all patients who were diagnosed with rectal cancer and underwent surgery for stoma closure at the Sixth Affiliated Hospital of Sun Yat-sen University (Guangzhou, China) between January 2014 and December 2017 in the present study. From January 2014 to December 2014, all skin wounds of patients were closed using the LS (the LS group). From January 2015 to December 2017, all skin wounds were closed using the GS (the GS group). Information of patient characteristics, intraoperative variables, and post-operative variables was collected, and SSIs were evaluated according to the Guidelines for the Centers for Disease Control and Prevention (CDC) [13].

### Surgical techniques and corresponding pictures

After successfully administering anesthesia, the stoma area was sterilized with iodophor and the stoma was closed using figure-eight sutures [14]. The skin was incised as close as possible to the circumference of the suture line and the bowel was dissected from the abdominal wall. Then, the small intestines on both sides of the stoma were anastomosed using a linear stapler or in a hand-sewn manner. The peritoneum and fascia were closed using interrupted absorbable sutures [15].

**Figure 1.**
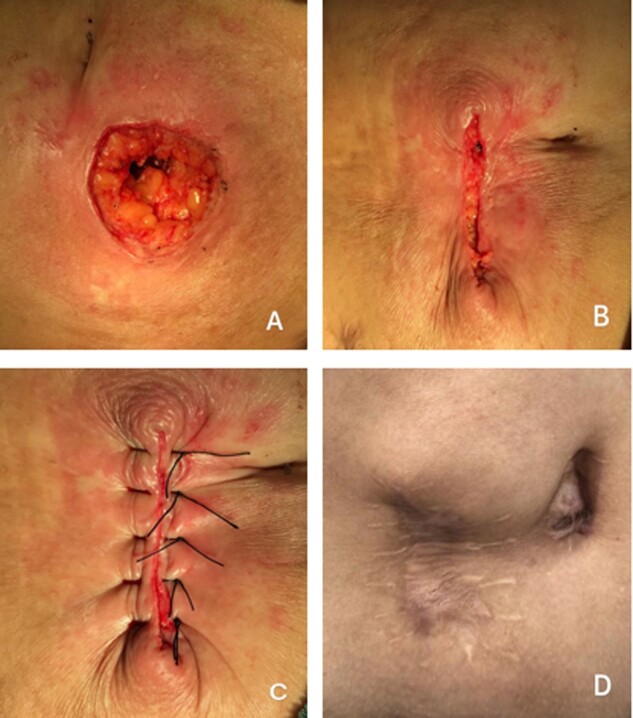
Diagrammatic representation of linear skin closure (A)–(C) and the photographs at 1 year after ileostomy closure (D). Stoma takedown and reconstruction were performed (A). The peritoneum and subcutaneous tissue were sutured (B) and skin was closed using unabsorbable sutures.

**Figure 2.**
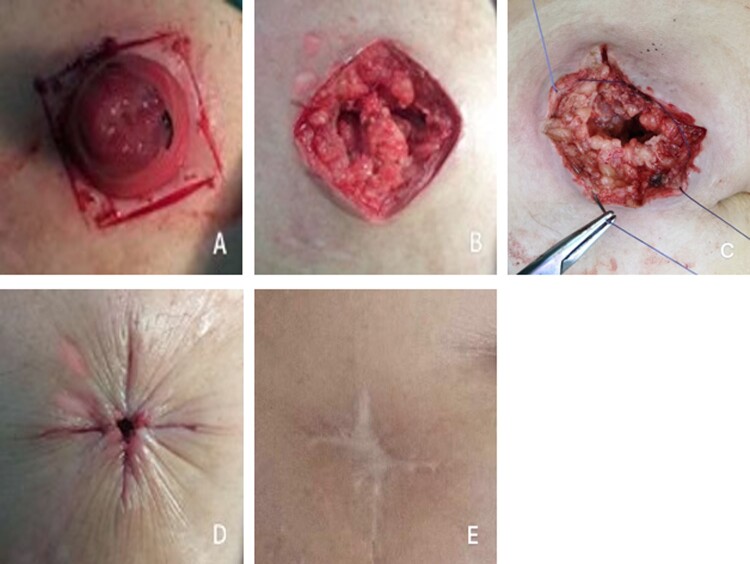
Diagrammatic representation of gunsight skin closure and the photographs at 1 year after ileostomy closure. A diamond incision was made in the skin with one of the stoma openings used as the internal incision edge (A). Stoma takedown and reconstruction were performed (B). After the fascia and peritoneum were sutured, circumferential subcuticular sutures (absorbable material) were placed at the wound edge, reducing the overall diameter of the defect and leaving a small residual hole in the skin (C) and (D). (E) Long-term healing of the wound.

For skin closure, after the muscle layer was sutured, the subcutaneous tissue was washed using warm physiological saline solution. In the LS group, the skin was sutured with three or four interrupted stitches using non-absorbable monofilament sutures (**Figure 1A–C**).The skin-closure steps in the GS group were performed as follows [9]. (i) A diamond incision was made in the skin using one of the stoma openings as the internal incision edge (**Figure 2A**). (ii) Stoma takedown and reconstruction were performed in the GS group in a manner similar to that in the LS group (**Figure 2B**). (iii) After the fascia and peritoneum were sutured, as in the LS group, circumferential subcuticular sutures (absorbable material) were placed at the wound edge, reducing the overall diameter of the defect and leaving a small residual hole in the skin (**Figure 2C and D**). The sutures in the subcutaneous layer and skin should not be too tight; ∼0.5 cm of space should be retained for drainage.

### Primary and secondary endpoints

The primary endpoint of this study was the rate of SSI according to the definition of the CDC [13, 16]. The diagnostic criteria of SSI [17] were based on the "Diagnostic criteria for nosocomial infections" by the Ministry of Health in 2001 (Trial) as the standard guidelines [18, 19].

The secondary endpoints were the post-operative length of hospital stay, incidence of post-operative bowel obstruction, and duration of the operation. Other factors that could affect the SSI rate, such as age, sex, body mass index, preoperative serum albumin level, duration of stoma, distance between the tumors and the anal margin, and anastomotic methods, were also evaluated.

### Statistical analysis

All statistical analyses were performed using SPSS (version 25.0 for Windows; SPSS, Chicago, IL, USA). Measurement data are expressed as the mean ± standard deviation or median (minimum, maximum). *T*-tests or Mann–Whitney rank-sum tests were used to compare the measurement data between the groups and logistic regressions were used to analyse the univariate and multivariate factors for SSI after the operation. Variables with *P* < 0.2 in the univariate analysis were selected for the multivariate analysis using logistic regression. Differences were considered statistically significant when *P* < 0.05.

## Results

A total of 233 patients were included in this study, including 130 patients who received LS and 103 patients who received GS. All patients received preoperative antibiotics and successfully underwent surgery. **Table 1** presents the differences in characteristics between patients with LS and GS. The mean age of the LS group was 56.8 ± 12.9 years and 80 (61.5%) patients who received LS were male. The GS group had a median age of 53.1 ± 11.6 years and 72 (69.9%) patients were male. The mean age of the GS group was significantly younger than that of the LS group (53.1 ± 11.6 vs 56.8 ± 12.9 years, *P* = 0.023). Hand-sewn anastomoses were performed in 39 (30%) of the LS group and 14 (14%) of the GS group (*P* = 0.003). The rate of SSI was significantly lower in the GS group (*n* = 2, 2%) than in the LS group (*n* = 16, 12%, *P* = 0.007). All patients with SSIs were treated with oral or intravenous antibiotics and wound dressings with dry gauze. The post-operative length of hospital stay was significantly shorter in the GS group (8.10 ± 3.15 days) than in the LS group (10.79 ± 5.44 days, *P* < 0.001) (**Table 1**). **Figure 1D** shows the long-term healing of the wound after the LS technique and **Figure 2E** shows the long-term healing of the wound after the GS technique.

**Table 1.** Characteristics of 233 patients who underwent loop ileostomy

Characteristic	LS group (*n* = 130)	GS group (*n* = 103)	*P*
Age (years)	56.8 ± 12.9	53.1 ± 11.6	0.023
Sex			0.332
Male	80 (61.5%)	72 (69.9%)	
Female	50 (38.5%)	31 (30.1%)	
Body mass index	22.0 ± 3.1	22.0 ± 3.0	0.852
Distance between tumors and the anal margin (cm)	6.2 ± 3.2	5.5 ± 2.1	0.057
Albumin (preoperative, g/L)	41.8 ± 3.9	42.5 ± 3.52	0.157
Duration of stoma (days)	156.4 ± 90.2	151.7 ± 61.0	0.653
Duration of surgery (min)	90.3 ± 51.0	96.1 ± 39.8	0.344
Hospital stay (days)	10.8 ± 5.4	8.1 ± 3.2	<0.001
Anastomotic method			
Mechanical	90 (69.2%)	89 (86.4%)	0.003
By hand	40 (30.8%)	14 (13.6%)	
Surgical-site infection			
Yes	16 (12.3%)	2 (1.9%)	0.007
No	114 (87.7%)	101 (98.1%)	
Bowel obstruction			
Yes	9 (6.9%)	11 (10.7%)	0.311
No	121 (93.1%)	92 (89.3%)	
			

LS, linear suture; GS, gunsight suture.

Age, body mass index, distance between tumors and the anal margin, albumin, duration of stoma, duration of surgery, and hospital stay are presented as mean number ± standard deviation; sex, anastomotic method, surgical-site infection, and bowel obstruction are presented as number of patients (percentage).

*T*-tests or Mann–Whitney rank-sum tests were used to compare the measurement data between groups, Differences were considered statistically significant when *P* < 0.05.

We assessed the risk factors for SSI from univariate and multivariate analyses (**Table 2**). Univariate analysis and multivariate analysis showed that GS (odds ratio = 0.212, *P* = 0.048) was an independent protective factor for SSI after stoma reversal.

**Table 2.** Univariate and multivariate logistic-regression model representing risk factors of SSI for 233 patients after operation

Characteristic	Infected	Uninfected	Univariate analysis	Multivariate analysis	
			*P*	HR (95% CI)	*P*
Age (years)	59.4 ± 9.4	54.9 ± 12.6	0.178	1.106 (0.969–1.066)	0.051
Sex (male:female)	12:3	143:75	0.262		
Body mass index	22.22 ± 3.60	21.98 ± 3.02	0.764		
Distance between tumors and anal margin	3.8 ± 1.0	2.7 ± 0.2	0.896		
Albumin	40.9 ± 2.8	42.2 ± 3.8	0.184	0.931 (0.798–1.087)	0.366
Prealbumin	0.3 ± 0.6	0.6 ± 4.3	0.891		
Duration of stoma (days)	175.9 ± 151.2	152.9 ± 71.3	0.279		
Anastomotic			0.338		
Mechanical	10 (66.7%)	169 (77.5%)			
Hand	5 (33.3%)	49 (22.5%)			
Suture technique			0.025	0.212 (0.046–0.097)	0.048
LS	16 (88.9%)	114 (53.0%)			
GS	2 (1.1%)	101 (47.0%)			
Duration of surgery (min)	99.2 ± 39.5	92.4 ± 46.9	0.583		

LS, linear suture; GS, gunsight suture.

Logistic regressions were used to analyse the univariate and multivariate factors for SSI after the operation. Variables including age, prealbumin, and suture technique with *P* < 0.2 in the univariate analysis were selected for the multivariate analysis using logistic regression. Differences were considered statistically significant when *P* < 0.05.

## Discussion

Patients with colorectal diseases, such as colorectal cancers, inflammatory bowel disease, and rectal trauma, usually need to undergo preventive ileostomy after colorectal surgery. Ileostomy reversal is a commonly planned operation in colorectal surgery [20] that is used to restore intestinal continuity after the primary wound heals. However, the most common type of complication after ileostomy closure is SSI, once infection occurs, which can seriously affect increase costs, prolong the hospitalization time, and affect the patient’s quality of life. The incidence rates reported domestically and internationally range from 2% to 41% [2]. Therefore, reducing the rate of SSI after ileostomy reversal is an urgent problem that remains to be solved; different kinds of suture techniques have been proposed with the goal of reducing the incidence of SSI. However, the ideal skin-closure technique has not yet been determined currently. Our study was conducted to compare the incidence of SSI between LS and GS retrospectively.

The ideal technique for peristomal incisions and closures should achieve the following: low rate of wound infections, minimal post-operative care, and good surgical access. Studies have shown that LS has a high SSI rate. Bacterial contamination of the stoma skin may be an important factor for the high infection rate of primary suture wounds. Simply administering antibiotics is not effective at preventing infections and delayed wound healing often occurs. In addition, LS makes circular incisions that form a linear wound that has high tension, which causes tension in the abdominal-wall skin and results in limited movement for patients, and obvious skin remains at the later stage of the wound-healing process. In addition, scarring will affect the aesthetics of the wound and comfort of the patient to varying degrees. Purse-string skin closures after stoma reversal were first introduced by Banerjee in 1997 [4] and led to a significantly reduced risk of SSI. Several studies have reported the SSI rate following ileostomy closure with LS and purse-string sutures for skin closure [21, 22]. In their study, Reid *et al[6].* found that the SSI rate of direct sutures was significantly higher than that of purse-string sutures (39% vs 7%; *P* = 0.05). In the study by Bell *et al[23]*, 11 patients were diagnosed with incisional SSI. In the study by Sathasivam *et al.*, the incidences of SSI were significantly different between the conventional linear-closure group and the purse-string-closure group (17 vs 3; *P* = 0.003). The Patient and Observer Scar Assessment Scoring scores in each group were significantly different between the two groups (65.30 vs 83.40; *P* = 0.012) [24]. In 2010, Lim *et al*. [9] introduced the gunsight-closure technique, which not only has a low SSI rate, but also provides improved access to allow wound drainage, simplifies wound management after surgery, and maintains a clean cosmetic effect [25]. Our study confirmed that, compared with LS, GS significantly reduces the wound-infection rate and shortens hospital stay. We believe that, for the GS group, retaining a 0.5-mm central pore can help to drain the exudation fluid and aid in the early detection of infections and administration of early treatment. In the rare event of wound infection, drainage is still in place to facilitate wound healing. However, compared with previous studies in this area, our study failed to include the wound-scar score or aesthetic results. With advancements in technology, patients increasingly pay attention not only to SSI, but also to the beauty of the skin and the appearance of the scar (Figures 1D and 2E), which is a limitation of our study. Our study shows that the duration of surgery was longer in the GS group than in the LS group. This could be attributed to the relatively sophisticated design of the GS and the learning curve needed to master this technique. Elaborate design of the initial incision is of paramount importance to achieve minimal tension and excellent approximation. Performed skillfully, the GS technique can provide excellent exposure and improved SSI and aesthetic outcomes. In the studies of Reid *et al.* [6] and Sajid *et al.* [7], the difference in the mean length of hospital stay was not significant between the two groups [undefined, 7]. In the present study, the mean hospital stay between the two groups did not show any significant difference. The SSI led to prolonged hospital stay due to the requirement for frequent debridement and specialized dressings. In the present study, all SSI were superficial incisional infections and were treated conservatively. In our study, the length of hospitalization stay in both groups was longer than the results of present studies, the reason for which may be that the protocol of our inpatient department for patients undergoing stoma reversal includes preoperative examinations and recovery of post-operative patients. It may take a long time to finish the preoperative examination and the defecation function should be regularly observed after surgery. Thankfully, along with the development of enhanced recovery after surgery, the post-operative hospitalization time and the hospitalization costs for patients have been reduced greatly.

Our findings also show that the proportion of suture methods between the two groups has changed significantly. In both groups, mechanical anastomosis was more prevalent than hand-sewn anastomosis but, in the GS group, the proportion of hand-sewn anastomoses was significantly higher compared with that in the LS group. The reason may be because, along with global development and advances in medical devices, the safety and effectiveness of anastomosis devices have greatly improved and thus mechanical anastomosis is widely used in clinical treatment.

As a retrospective analysis, our study has some shortcomings. There were no comparisons of post-operative wound-pain scores, scar scores, or the cosmetic effects between the two groups. A prospective randomized–controlled study will be conducted to further compare the two techniques in the future.

In general, GS significantly reduced the incidence of SSI and shortened the length of hospital stay after reversal of ileostomy. Therefore, we recommend that GS could be applied for skin closure after ileostomy reversal.

## Conclusions

This retrospective study shows that, compared with the LS technique, the GS technique can significantly decrease the rate of SSI and shorten the length of hospital stay after operation. As a result, the GS technique should be recommended for wound closure following ileostomy reversal. Large randomized–controlled trials are needed to confirm these findings.

## Authors’ contributions

C.K.L. performed the statistical analyses and interpretation, and drafted the manuscript. W.W.L. performed the statistical analyses and drafted the manuscript. H.M.W., W.T.G., and X.S.Q. participated in its design and coordination. J.Z., W.B.Z., and Y.L. collected information. H.W. conceived of the study. R.K.H. conceived of the study and participated in its design and coordination. All authors read and approved the final manuscript.

## Funding

The study was supported by the Guangzhou Science and Technology Plan Project [No. 201704020059 and 201803010074] and National Key Clinical Discipline.
